# Incubation temperature affects the immune function of hatchling soft-shelled turtles, *Pelodiscus sinensis*

**DOI:** 10.1038/srep10594

**Published:** 2015-06-01

**Authors:** Wei Dang, Wen Zhang, Wei-Guo Du

**Affiliations:** 1 Hangzhou Key Laboratory of Animal Adaptation and Evolution, Hangzhou Normal University, Hangzhou, 310036, China; 2 Key Laboratory of Animal Ecology and Conservation Biology, Institute of Zoology, Chinese Academy of Sciences, Beijing, 100101, China

## Abstract

Identifying how developmental temperature affects the immune system is critical for understanding how ectothermic animals defend against pathogens and their fitness in the changing world. However, reptiles have received little attention regarding this issue. We incubated eggs at three ecologically relevant temperatures to determine how incubation temperature affects the immune function of hatchling soft-shelled turtles, *Pelodiscus sinensis*. When exposed to bacterial infections, hatchlings from 24 °C had lower cumulative mortalities (55%, therefore, higher immunocompetence) than those from 28 °C (85%) or 32 °C (100%). Consistent with higher immunocompetence, hatchlings from low incubation temperature had higher IgM, IgD, and CD3γ expressions than their counterparts from the other two higher incubation temperatures. Conversely, the activity of immunity-related enzymes did not match the among-temperature difference in immune function. Specifically, enzyme activity was higher at intermediate temperatures (alkaline phosphatase) or was not affected by incubation temperature (acid phosphatase, lysozyme). Our study is the first to provide unequivocal evidence (at the molecular and organismal level) about the significant effect of incubation temperature on offspring immunity in reptiles. Our results also indicate that the reduced immunity induced by high developmental temperatures might increase the vulnerability of reptiles to the outbreak of diseases under global warming scenarios.

The survival of most animals is threatened by parasites and pathogens that are widespread in the natural environments. It was recently revealed that pathogen outbreaks have caused a global decline in amphibians and are expected to affect reptiles^1^,^2^. More seriously, this ecological disaster may be worsen under the background of climate change, because altered conditions reduce the defenses of vertebrates against pathogens^3^. The immune system is a defense mechanism that protects organisms against parasitic and pathogenic infections, and it is important for the survival of animals in an infectious environment. Apart from survival, immunity is also associated with behavioral and life-history traits, such as mate selection, reproductive output, and growth, because the immune response is energetically costly and potentially competes with other life-history parameters for limited resources^4^. Therefore, it is critical to understand how the immune system responds to environmental changes to determine the effectiveness of vertebrate defenses against pathogens and their fitness in the changing world.

The embryonic development of oviparous vertebrates is completed outside of the mother’s body. Thus, immunity is determined by both genetic and environmental factors, with the developmental environment of the eggs representing an important influence^5^,^6^. Large numbers of studies have focused on how temperature affects embryonic development and hatchling traits in reptiles and birds in recent decades^7^,^8^. Yet, the effect of incubation temperature on immune function has received limited attention. Recent studies on birds have indicated that temperature affects the immune function of hatchings, with decreased immunocompetence at lower temperatures (e.g., 35 °C) compared to higher temperatures (e.g., 37 °C)^5^,^6^. However, just one study has investigated how temperature affects offspring immunity in reptiles; namely, the map turtles (*Graptemys ouachitensis* and *Graptemys pseudogeographica*)^9^. In many turtles, eggs incubated at lower temperatures tend to produce more males, while those incubated at higher temperatures produce more females^10^,^11^,^12^,^13^. The study on *G. ouachitensis* indicated that males produced by low incubation temperatures have higher immunocompetence than females produced by high incubation temperatures^9^. Thus, temperature and sex have mixed effects in map turtles with temperature-dependent sex determination (TSD). Yet, information is required on whether hatchling immunity is influenced by incubation temperature, hatchling sex, or a combination of the two. Therefore, there is much debate over the extent to which incubation temperature impacts offspring immunity among species, meriting further studies to clarify this issue.

The development of strong innate and acquired immunity represents an effective strategy for animals to resist diseases in their habitats^4^. Innate immunity is nonspecific, constitutively expressed, and may be particularly important to the fitness and life history of an animal in its natural habitat, as it might determine the survival of an animal on its first encounter with a disease. Thus, a successful innate response may help avoid a costly antigen-specific response of acquired immunity. For example, lysosomal hydrolytic enzymes (e.g., lysozyme and acid phosphatase) are vital factors in innate immunity, and may kill bacteria or digest pathogens^14^. In addition, innate immunity responses stimulate the adaptive immune system. Humoral and cellular immune responses result in antibody production by bursa dependent lymphocyte (B) cells and cellular immunity by thymus-derived (T) cells. Consequently, bacteria are usually killed by these two responses. The enzymes of alkaline phosphatase, immunoglobulin M (IgM), and IgD produced by B cells are critical in the humoral immune response to infectious pathogens^15^,^16^. In addition, co-stimulatory molecules, such as CD3γ and CD9, are important in the process of cell-mediated immunity^17^,^18^,^19^. Exploring the effect of temperature on the expression of these immunity-related enzymes and genes would enhance our understanding about the proximate mechanisms by which developmental temperature affects offspring immunity in animals.

In this study, we aim to determine the effect of incubation temperature on the immune function of hatchling soft-shelled turtles, *Pelodiscus sinensis*. The hatchling sex of *P. sinensis* is determined genetically (genetic sex determination, GSD) rather than being influenced by incubation temperature (TSD)^20^. We thus use the Chinese soft- shell turtle as the subject of this study to avoid the confounding effects of incubation temperature and sex on offspring immunity. We incubated *P. sinensis* eggs at three temperatures that span the range of temperatures experienced by the eggs in natural nests. The hatchlings from these thermal treatments were exposed to bacterial infections and mortality was determined over a 1-week period. By analyzing the relationship between incubation period and the mortality of hatchlings, we aim to determine how incubation temperature influences immune function. To identify the underlying mechanism of temperature effects on offspring immunity, we determined the activity of specific immunity-related enzymes (such as lysozyme, acid phosphatase, and alkaline phosphatase) and the regulation of specific immune genes (including IgM, IgD, CD3γ, and CD9). Thus, we tested the hypothesis that the activity of these enzymes would increase and that the expression of these immune genes would become upregulated in hatchlings that had high immune function.

## Results

### Immunity

**Figure 1:**
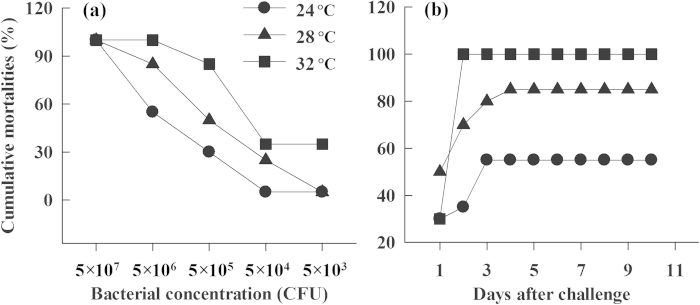
Effects of incubation temperature on the mortality of hatchling turtles (***P. sinensis***) exposed to bacterial infection. (**a**) Hatchling mortality at different concentrations of bacteria, (**b**) temporal variation in hatchling mortality at a bacterial concentration of 5 × 10^6^ CFU. Hatchlings from eggs incubated at low temperatures had lower mortality than those incubated at high temperatures.

After being challenged with a concentration gradient of the pathogen *Aeromonas hydrophilia* TL1 from 5 × 10^3^ to 5 × 10^7^ Colony-Forming Units (CFU), all hatchlings from all three incubation temperatures died at the concentration of 5 × 10^7^ CFU, and had similar cumulative mortalities at the concentration of 5 × 10^4^ CFU (*G* = 3.68, *df* = 2, *P* > 0.05). However, the cumulative mortalities differed at a concentration of 5 × 10^3^ CFU (*G* = 8.21, *df* = 2, *P* < 0.05), 5 × 10^5^ CFU (*G* = 21.40, *df* = 2, *P* < 0.005), and 5 × 10^6^ CFU (*G* = 14.68, *df* = 2, *P* < 0.001) (Fig. 1a). On exposure to the 5 × 10^6^ CFU TL1 challenge, hatchlings from eggs incubated at 24 °C (55%) had lower cumulative mortalities than those incubated at 28 °C (85%) and 32 °C (100%) (Fig. 1b). Hatchlings from eggs incubated at 24 °C had the highest LD50 (1.496 × 10^6^), followed by those incubated at 28 °C (LD50: 3.403 × 10^5^) and 32 °C (LD50: 2.942 × 10^4^).

### Enzyme activity

**Figure 2:**
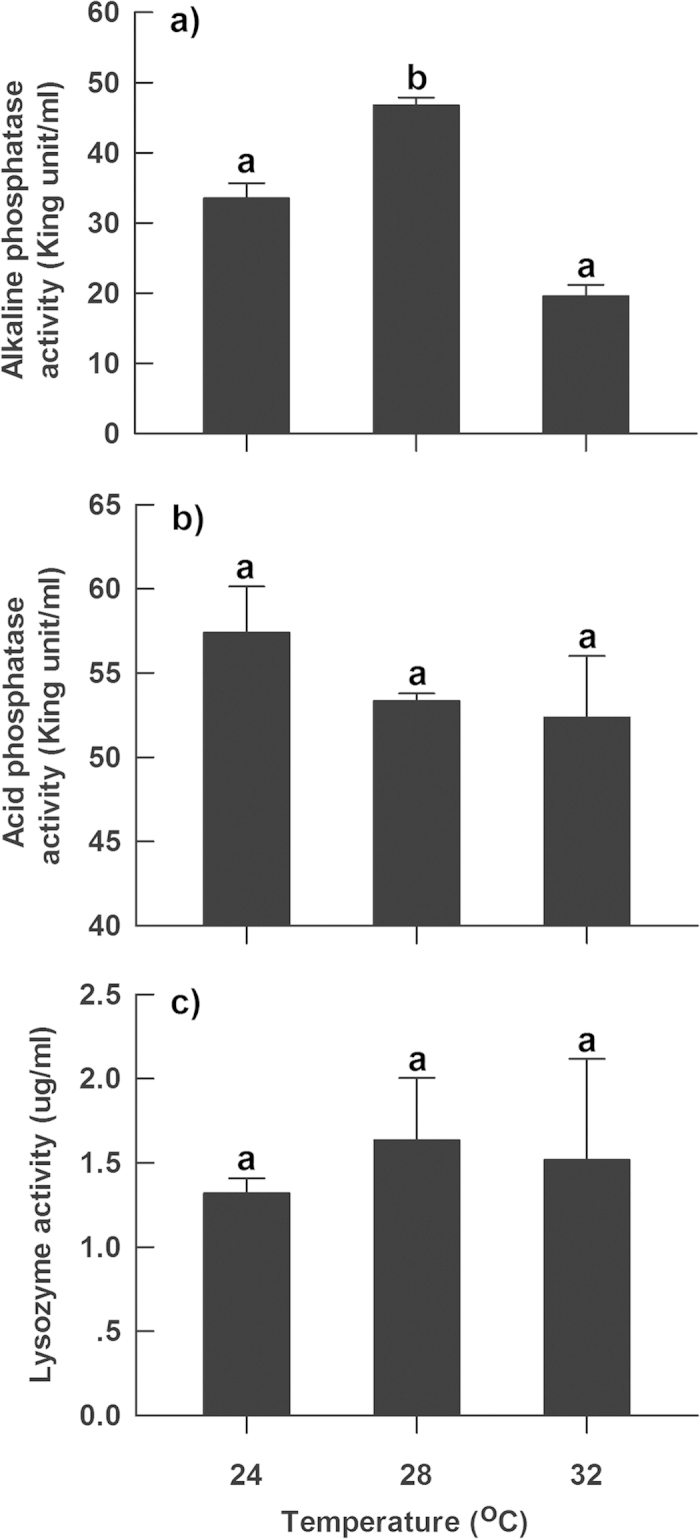
The activity of immunity-related enzymes in hatchling turtles (***P. sinensis***) from eggs incubated at different temperatures. Graphs show the mean values ± 1 SE. Means with different letters above the error bars are statistically different (Tukey’s test).

The activity of serum alkaline phosphatase was highest and lowest in hatchlings from eggs that had been incubated at 28 °C and 32 °C, respectively, and intermediate for those incubated at 24 °C (*F*_2,6_ = 64.94, *P* < 0.001) (Fig. 2a).In contrast, the activity of serum acid phosphatase and lysozyme was similar among hatchlings from the three different incubation treatments (*F*_2,6_ = 3.03, *P* = 0.123; *F*_2,6_ = 0.27, *P* = 0.775) (Fig. 2b,c).

### Immune gene expression

**Figure 3:**
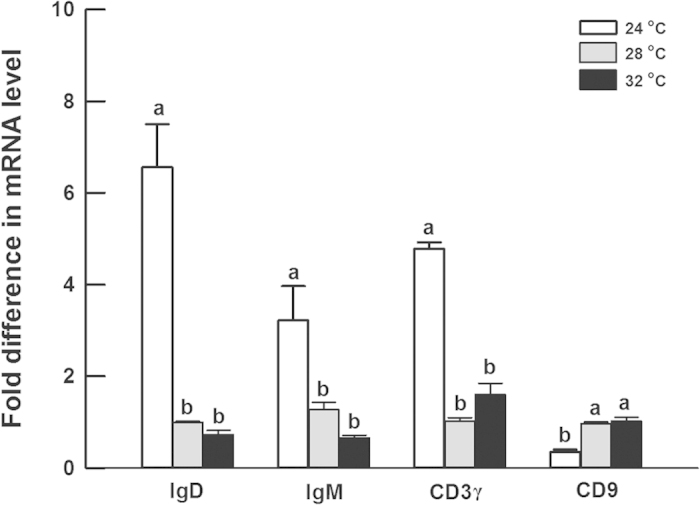
The expression of immune genes in hatchling turtles (***P. sinensis***) from eggs incubated at different temperatures. Most immune genes of hatchlings from low temperatures were upregulated, compared with those from high temperatures. Graphs show mean values ± 1 SE. Means with different letters above the error bars are statistically different (Tukey’s test).

The expression levels of all immune genes (including IgD, IgM, CD3γ, and CD9) measured in hatchlings were significantly affected by incubation temperature (IgD: *F*_2,6_ = 36.6, *P* < 0.001; IgM: *F*_2,6_ = 9.1, *P* < 0.05; CD3γ: *F*_2,6_ = 142.4, *P* < 0.0001; CD9: *F*_2,6_ = 96.3, *P* < 0.0001). Compared to hatchlings from eggs incubated at 28 °C, the expression level of IgD, IgM, and CD3γ was 6.6-fold, 3.2-fold, and 4.8 fold higher, respectively, in hatchlings from eggs incubated at 24 °C, whereas that of CD9 was significantly lower (Fig. 3). In contrast, the expression level of the four genes in hatchlings from eggs incubated at 32 °C was similar to that of those from 28 °C (Fig. 3).

## Discussion

Our results indicate that incubation temperature significantly affects immune function, in addition to associated biochemical and molecular processes, including the activity of immunity-related enzymes and the expression of immune genes. While several studies have found that incubation temperature may affect immune function of avian offspring^5^,^6^, similar studies are rare in reptiles. Our study provides unequivocal evidence that incubation temperature affects immune function and associated biochemical and molecular processes in GSD reptiles. Freedberg *et al.* (2008)^9^ found that incubation temperature significantly affected immunocompetence in one TSD reptile (*G. ouachitensis*), but not in another one (*G. pseudogeographica*), and speculated that the different immunocompetence was more likely attributed to incubation temperature than sex. This speculation is verified by our study since sex was disassociated from temperature in GSD reptiles. In addition, male vertebrates may have a higher risk of infection, and therefore have a greater need for increased innate immunity than females^9^. Our results give support to this hypothesis as well as an adaptive explanation for TSD in the light of Charnov-Bull model^21^, that TSD may have evolved to allow the production of males at temperatures that enhance immunity, a trait that may be more important for male fitness^9^.

Immunity-related genes and enzymes may be critical towards understanding how incubation temperature affects offspring immune function. IgD and IgM are members of the immunoglobulin classes, and are co-expressed on the membranes of most B cells to form mature antibodies^22^. Thus these immunoglobulins may provide a first line of defense against microbial infection, together with innate immunity factors^23^. In this study, the expression of genes involved in acquired immunity matched the temperature difference in immune function, with higher expression being recorded at lower temperatures compared to higher temperatures (Fig. 1 and Fig. 3). The two markers of acquired immunity (CD3γ and CD9) showed different expression patterns. The expression of CD3γ, which is the signal transporter for T cells^17^, match the temperature-induced difference in immune function. In contrast, the expression of CD9, which is associated with major histocompatibility complex class II molecules at the plasma membrane^18^,^19^, demonstrated opposite patterns to the observed differences in immune function. Given that CD9 does not modulate CD3-mediated signaling^24^, it is likely that low incubation temperatures stimulated the signal pathway associated with CD3 rather than CD9. Lysozyme, acid phosphatases, and alkaline phosphatases are all hydrolase enzymes involved in immunity. Lysozyme may hydrolyze the 1,4-beta-linkages of bacterial cell walls to lethally damage to bacteria^14^. Acid and alkaline phosphatases may catalyze the hydrolysis of various phosphate-containing compounds at acidic pHs and act as transphosphorylases at alkaline pHs^25^. However, in contrast to our expectation, there was no positive relationship between enzyme activity and immune function in hatchling *P. sinensis* (Fig. 2). The inconsistence between enzyme activity and immune function implies that the expression of these immune enzymes might not be modulated by incubation temperatures during embryonic development. Instead, their expression may be responsive to environmental stress and pathogen infection faced by hatchlings, which has been demonstrated in other species^26^,^27^,^28^.

In addition to immunity-related genes and enzymes, hormones may also be important for the development of immunity function. Both testosterone and dihydrotestosterone (DHT) tend to impair immunological responses, whereas estradiol tends to enhance immunological function^29^,^30^. Our study did not directly address how temperature-induced hormone changes may affect immune development in turtles, although a similar physiological mechanism seems plausible.

The formation of a mature immune system is a long-term dynamic process from a fertilized egg to an adult. Our study focused on how temperature during embryonic development affects the initial phase of immune system formation. A number of other studies have shown that temperature also affects the immune function of individuals after hatching. For example, acute and chronic cold stress may enhance the expression of immunoglobulin and cytokine involved in the immune system of birds^31^. In addition, a study of *Nile tilapia* juvenile fish indicated that suitable temperature may increase the concentration of hematological parameters (e.g., white blood cells and hemoglobin) that have functional immune roles to strengthen non-specific immunity^32^.

There is increasing evidence that the developmental environment may significantly modify the immune function of hatchings in oviparous vertebrates like reptiles and birds^6^,^9^. The importance of such studies should be emphasized for at least two reasons. First, many studies have demonstrated that the developmental environment induces significant phenotypic variations in hatchling traits (e.g., body size and locomotor performance), which are potentially related to offspring fitness^20^,^33^,^34^,^35^. However, these studies have rarely gone on further to actually demonstrate the existence of this relationship because of logistical difficulties in evaluating fitness (survival and reproduction), especially for long-lived species, such as turtles but see^36^. Immunity function may determine the survival of offspring and, thus, provide an important direct index of offspring fitness, which could be used to understand the role of developmental plasticity in adaptive traits by future studies. Second, there is increasing research focus on how global warming would affect the fate of ectothermic vertebrates like amphibians and reptiles^37^,^38^. Global warming may shorten the incubation period^39^ and, therefore, reduce egg mortality and increase neonate survival^40^. In contrast, our experimental results indicate that reduced immunity due to global warming causing higher temperatures during development might increase the vulnerability of reptiles to outbreaks of parasitic and pathogenic diseases^1^.

## Methods

### Ethics statements

This research was performed according to the NIH *Guide for the Principles of Animal Care*. The protocols and study were approved by the Animal Ethics Committee at the Institute of Zoology, Chinese Academy of Sciences (Permit Number: IOZ14001).

### Study species and bacterial strain

The Chinese soft-shelled turtle (*P. sinensis*) is distributed in mainland China and southeastern Asia^41^, and has been widely cultured in China for food. From May to August, female *P. sinensis* individuals lay multiple clutches of eggs (mean clutch size = 20)^42^. Incubation temperatures significantly affect embryonic development (e.g., developmental rate, embryonic utilization of energy, and hatching success) and hatchling traits (e.g., body sizes, locomotor performance, and post-hatching growth)^20^,^33^,^43^.

The pathogen strain, *A. hydrophilia* TL1, was isolated from diseased Chinese soft-shelled turtles at a turtle farm in the Zhejiang Province of China, and was genetically identified by 16s rRNA gene sequence analysis (GenBank accession no. KJ743719) according to Zhang and Sun’s method^44^. The pathogenicity of strain TL1 was examined in the laboratory using juvenile Chinese soft-shell turtles (~7 g) purchased from the farm. It was found that 5 × 10^6^ CFU TL1 caused 80% mortality at a water temperature of 25 °C. The strain TL1 was cultured in Luria-Bertani lysis broth (LB) medium^44^ at 28 °C.

### Egg collection and incubation

A total of 450 freshly laid fertilized eggs of *P. sinensis* (identified by a white patch on the shell surfaces, mean egg mass = 5.35 g) were collected from a private hatchery in Zhejiang Province, eastern China. The eggs were weighed to an accuracy of ±1 mg using an electronic balance (Mettler Toledo AB135-S, Germany), and were placed in plastic containers (25 × 20 × 10 mm) filled with moist vermiculite of −220 kPa (1 g water/1 g vermiculite) according to Du *et al.*^45^ Eight eggs were placed in each container. The containers were placed into one of three incubators (Ningbo Life Science and Technology Ltd, China), in which the temperature was set to 24 °C, 28 °C, and 32 °C, respectively, to cover the temperature range experienced by the eggs in artificial nests in outdoor enclosures^33^. The maternity of these eggs was unknown, originating from about 22 females based on the clutch size (mean clutch size = 20) of this species. Thus, the eggs were randomly assigned to the three different treatments to minimize maternal effects. It was not possible to examine the potential clutch effects on our results, due to the uncertainty of maternal identity in these eggs. However, such effects would have been largely reduced by the relatively large number of clutch origins and random assignment of eggs among treatments. We moved the containers among shelves according to a predetermined schedule to minimize any effects of thermal gradients inside the incubators.

### Incubation period

Toward the end of incubation, the jars were monitored once a day for newly emerging hatchlings. The number of days that elapsed between the beginning of incubation and the emergence of the hatchlings was recorded as the incubation period. After emergence, the hatchlings were maintained in the jar until the yolk had been entirely absorbed (usually within two days). The turtles were then kept in separate cages in a temperature-controlled room at 28 ± 1 °C and with a 12-h light/12-h dark cycle according to Du *et al.*^45^.

### Immunocompetence test

To determine the immunocompetence of hatchling turtles, we measured the survival rate of the hatchlings against bacterial infection (*A. hydrophilia* TL1) according to Hu *et al.*^46^. Hatchlings (n = 100) from each incubation temperature treatment were randomly assigned to five groups (20 individuals per group) with different bacterial challenge treatments: 5 × 10^7^, 5 × 10^6^, 5 × 10^5^, 5 × 10^4^, and 5 × 10^3^ CFU of TL1. TL1 was injected into the turtles intraperitoneally, and monitored for mortality up to 10 days post-challenge.

### Tissue collection

Three newly hatched Chinese soft-shelled turtles from each temperature treatment were randomly sacrificed to collect blood and liver samples for subsequent biochemical analysis as previously described^47^. Briefly, the blood was incubated at 37 °C for 1 hour, and then centrifuged (5000 rpm/min) at 4 °C for 10 min to collect serum. The liver was removed aseptically and frozen in liquid nitrogen.

### Acid and alkaline phosphatase assay

Serum acid phosphatase activities were measured by King and Jagatheesan’s method^48^, and serum alkaline phosphatase activities were measured by Kind and King’s method^49^. Commercially accessible kits (Nanjing Jiancheng Bioengineering Institute, Nanjing, China) were used. The activities of acid and alkaline phosphatases were determined by catalyzing the splitting of phosphoric acid from para-nitrophenyl phosphate (pNPP) to form free phenol. In the alkaline medium and in the presence of the oxidizing agent potassium ferricyanide, free phenol reacted with 4-aminoantipyrene to produce a red colored compound. This compound was estimated at 520 nm against a reagent blank. In brief, the alkaline phosphatase assay was operated as follows. We mixed 5 μl serum with 50 μl pNPP (20 mM) as the substrate and 50 μl carbonate buffer (0.1 M, pH10) containing 5 mM 4-Aminoantipyrene (Sangon, Shanghai, China). The mixture was incubated at 37 °C for 15 min, and then 150 μl chromogenic reagent (5 mM potassium ferricyanide, 250 mM boric acid) was added. The OD520 was recorded. The acid phosphatase assay was operated with citrate buffer (0.2 M, pH 5.5) as the replacement for carbonate buffer and 75 μl sodium hydroxide (7.5 M) was added with 75 μl chromogenic reagent. Double diluted water and phenol standard working solution (1 mg/ml) (Sangon, Shanghai, China) were used separately as the blank and standard, respectively. Optical density calculations were expressed as 1 mg phenol liberated by 100 ml serum for 15 min at 37 °C.

### Serum lysozyme assay

The serum lysozyme activities were measured by Turbidometric assay based on Ghafoori *et al.*^50^ with slight modification. Briefly, lyophilized *Micrococcus lysodeikticus* was suspended in PBS (pH 7.4) to 250 μg/ml. The bacteria solution and serum were incubated at 37 °C for 5 min. Then, 100 μl bacteria solution and 10 μl serum were immediately mixed, and the transmittance at 530 nm was recorded at 5 and 125 seconds. Hen egg white lysozyme (2.5 μg/ml) (Sangon, Shanghai, China) was used as the standard.

### Immune gene expression

Immune gene expression analysis was assessed in cDNA reversely transcripted by mRNA isolated from the tissues as previously described^51^. In briefly, we collected 30 mg of liver from each turtle, and extracted total RNA using TRIzol reagent (Ambion) (Invitrogen, Carlsbad CA, USA). One microgram of total RNA was treated with the gDNA Eraser kit (Takara, Dalian, China) and used for cDNA synthesis with the PrimeScript™ RT reagent Kit (Perfect Real Time). Quantitative real time reverse transcriptase-PCR (Qrt-PCR) was carried out in a C1000TM thermal cycler (Bio-Rad, Hercules CA, USA) using the iTaq Universal SYBR Green Supermix Kit (Bio-Rad, Hercules CA, USA). Each assay was performed in triplicate and programmed as follows: 95 °C for 3 min, then 40 cycles at 95 °C for 10 seconds, and 59 °C for 10 seconds, and 72 °C for 30 seconds. A negative control without a template was included in each assay. The melting curve analysis of the amplification products was performed at the end of each PCR to confirm that only one product was amplified and detected. The primers used to amplify the β-actin, IgD, IgM, CD3γ, and CD9 genes were designed according to published Chinese soft-shelled turtle sequences (Table 1).

### Data analysis

Statistical analyses were performed with SPSS 18.0 software (SPSS Inc., USA). The normality of distributions and the homogeneity of variances were tested with a Kolmogorov-Smirnov test and a Bartlett’s test, respectively. Analysis of variance (ANOVA) was used to determine the influence of incubation temperature on incubation period, while analysis of covariance (ANCOVA) was used to determine the influence of incubation temperature on hatchling mass, using initial egg mass as the covariate. We used the G-test to detect the among-treatment difference in cumulative mortality and probit analysis to calculate the median lethal dose (LD50) of hatchlings exposed to the bacterial infection. Acid and alkaline phosphatase activity (King unit/100 ml) were both calculated as :[(serum OD value – blank OD value) / (standard OD value – blank OD value)] × Standard concentration (0.1 mg/ml) × 100 ml^44^,^45^. Lysozyme activity was calculated according to the following formula: lysozyme content (μg/ml) = [(UT_2_-UT_0_)/(ST_2_-ST_0_)] × Standard concentration (2.5 μg/ml), where UT_2_ = the transmittance of serum at 5 seconds, UT_0_ = the transmittance of serum at 125 seconds, ST_2_ = the transmittance of hen egg lysozyme at 5 seconds, and ST_0_ = the transmittance of hen egg lysozyme at 125 seconds^46^. The expression levels of the IgD, IgM, CD3γ, and CD9 genes were analyzed using the 2^−ΔΔCT^ method^52^, and are presented in terms of relative mRNA (mean ± SE). One-way ANOVA was used to determine the difference in enzyme activity and immune gene expression. A Tukey post hoc multiple comparisons test was used to detect differences among treatments.

## Additional Information

**How to cite this article**: Dang, W. *et al.* Incubation temperature affects the immune function of hatchling soft-shelled turtles, *Pelodiscus sinensis*. *Sci. Rep.* **5**, 10594; doi: 10.1038/srep10594 (2015).

## Figures and Tables

**Table 1:** Primers used in Qrt-PCR.

**Gene**	**Genebank accession number**	**Primers**
IgD	FJ605149	F: 5′ - TGGGAACAAGGCACCAGATTT -3′
		R: 5′ - TTCGCAGACAAGAGTCAAGGA -3′
IgM	FJ605150	F: 5′ - GCTTATCCCACCGACCTTTG -3′
		R: 5′ - TCATCTCCTCGCTCCCACTC -3′
CD3γ	GU168571	F: 5′ - ATGAGGAGGGCGAGCACC - 3′
		R: 5′ - GCAAACGCATTACAAGGAGGA - 3′
CD9	FJ975143	F: 5′ - TATCCTCTGCGTCCCGTCC - 3′
		R: 5′ - CAAACCGAAGCCATAGTCCAA - 3′
β-actin	EU727174	F: 5′ - TGTTACCCATACTGTGCCCATC - 3′
		R: 5′ - TAGCCATCTCCTGTTCAAAATCC -3′
